# The influence of water temperature on sockeye salmon heart rate recovery following simulated fisheries interactions

**DOI:** 10.1093/conphys/cox050

**Published:** 2017-08-22

**Authors:** Tanya S. Prystay, Erika J. Eliason, Michael J. Lawrence, Melissa Dick, Jacob W. Brownscombe, David A. Patterson, Glenn T. Crossin, Scott G. Hinch, Steven J. Cooke

**Affiliations:** 1 Department of Biology, Dalhousie University, Halifax B3H 4R2, Canada; 2 Fish Ecology and Conservation Physiology Laboratory, Department of Biology and Institute of Environmental Science, Carleton University, Ottawa K1S 5B6, Canada; 3 Department of Ecology, Evolution and Marine Biology, University of California, CA 93106, USA; 4 Fisheries and Oceans Canada, School of Resource and Environmental Management, Simon Fraser University, Burnaby V2R 5B6, Canada; 5 Department of Forest and Conservation Sciences, University of British Columbia, Vancouver V6T 1Z4, Canada

**Keywords:** Cardiac, climate change, exhaustive exercise, fisheries, Pacific salmon, temperature

## Abstract

Selective harvest policies have been implemented in North America to enhance the conservation of Pacific salmon (*Oncorhynchus* spp.) stocks, which has led to an increase in the capture and release of fish by all fishing sectors. Despite the immediate survival benefits, catch-and-release results in capture stress, particularly at high water temperatures, and this can result in delayed post-release mortality minutes to days later. The objective of this study was to evaluate how different water temperatures influenced heart rate disturbance and recovery of wild sockeye salmon (*Oncorhynchus nerka*) following fisheries interactions (i.e. exhaustive exercise). Heart rate loggers were implanted into Fraser River sockeye salmon prior to simulated catch-and-release events conducted at three water temperatures (16°C, 19°C and 21°C). The fisheries simulation involved chasing logger-implanted fish in tanks for 3 min, followed by a 1 min air exposure. Neither resting nor routine heart rate differed among temperature treatments. In response to the fisheries simulation, peak heart rate increased with temperature (16°C = 91.3 ± 1.3 beats min^−1^; 19°C = 104.9 ± 2.0 beats min^−1^ and 21°C = 117 ± 1.3 beats min^−1^). Factorial heart rate and scope for heart rate were highest at 21°C and lowest at 16°C, but did not differ between 19°C and 21°C. Temperature affected the initial rate of cardiac recovery but not the overall duration (~10 h) such that the rate of energy expenditure during recovery increased with temperature. These findings support the notion that in the face of climate change, efforts to reduce stress at warmer temperatures will be necessary if catch-and-release practices are to be an effective conservation strategy.

## Teaser

This study describes sockeye salmon heart rate recovery profiles after a fisheries catch-and-release simulation in 16°C, 19°C and 21°C water. Warmer temperatures increased the peak heart rate, scope for heart rate, factorial heart rate and the initial rate of energy expenditure, though the overall recovery duration was consistent across treatments.

## Introduction

Catch-and-release practices have been implemented by commercial, recreational and indigenous fisheries sectors to enhance the conservation of Pacific salmon (*Onchorhynchus* spp.) populations (Department of Fisheries and Oceans, 2005), whereby more plentiful stocks are harvested while at-risk and non-targeted species are released. Despite the potential conservation value of catch-and-release fisheries practices, capture is stressful to fish (Davis and Olla, 2001; Arlinghaus *et al.*, 2007; Davis *et al.*, 2011). The behavioral and physiological responses to fisheries interactions are highly context dependent, varying among species, populations and even sexes (reviewed in Raby *et al.*, 2015a; Patterson *et al.*, 2016). Stressors include air exposure, handling, physical injury and exhaustive exercise, for which the resulting physiological disturbances tend to be magnified at warmer temperatures (Chopin and Arimoto, 1995; Murphy *et al.*, 1995; Olla *et al.*, 1998; Davis and Olla, 2001; Cooke and Suski, 2005; Gale *et al.*, 2013). These stressors can lead to ionic and osmotic dysregulation (Donaldson *et al.*, 2010, 2011; Gale *et al.*, 2011; Robinson *et al.*, 2013), increased use of anaerobic metabolism (Raby *et al.*, 2015b; Eliason *et al.*, 2013a,b), an increase in energy mobilization and changes in cardiovascular performance (Cooke *et al.*, 2003; Donaldson *et al.*, 2010; Raby *et al.*, 2015b). Negative impacts on post-release behavior include loss of equilibrium (Gale *et al.*, 2011), impaired swimming performance (Cooke *et al.*, 2000; Davis, 2005; Danylchuk *et al.*, 2007; Schreer *et al.*, 2005; Donaldson *et al.*, 2012), and reduced predator avoidance abilities (Mesa *et al.*, 1994; Lima and Dill, 1990). The magnitude of the stressor will ultimately determine its effects once fish are released, ranging from no immediate or long-term deleterious effects to lethal effects (Davis, 2002; Cooke and Schramm, 2007). Extreme temperatures have been implicated in the failure of salmon to complete migration (Farrell *et al.*, 2008; Crossin *et al.*, 2008) and has contributed to declines in spawner abundance across many populations (Martins *et al.*, 2011; Hinch *et al.*, 2012). As Pacific salmon are a semelparous species, the outcome of failed migration equals zero individual fitness and reduced population growth.

Previous studies investigating fish responses to catch-and-release have primarily focused on the immediate behavioral and physiological impacts (e.g. reflex impairment scores, blood parameters; reviewed in Cooke *et al.*, 2013). Many of these studies explored the relationship between fisheries interactions and subsequent behavior and survival using electronic tracking techniques (Donaldson *et al.*, 2008, 2011, 2012; Raby *et al.*, 2013). In addition, studies have looked at snapshots of physiological recovery, using blood or gill samples collected 1–3 times per individual to avoid harming the fish (Patterson *et al.*, 2004; Shrimpton *et al.*, 2005; Gale *et al.*, 2011). In contrast, the real time physiological stress response throughout the period of capture, release and recovery has not been well resolved. Fisheries capture induces an acute stress response, which is primarily mediated by the release of epinephrine, norepinephrine and cortisol from the adrenocortical tissues of the head kidney (Norris and Carr, 2006). Due to technical limitations, it is not possible to directly monitor the continual secretion of the stress hormones that mediate a fisheries interaction stress response. However, since one of the many effects of these hormones is to increase heart rate (*f*_H_) and energy mobilization, remote measurement of *f*_H_ is a proxy for those effects (Cooke *et al.*, 2002, 2016; Clark *et al.*, 2010). In fish, *f*_H_ alone cannot be used to calculate oxygen consumption (given that oxygen uptake = *f*_H_ × stroke volume × arteriovenous oxygen extraction; Thorarensen *et al.*, 1996; Priede and Tytler, 1997; Farrell, 1993). Nevertheless, past research conducted on various fish species, including salmonids, has demonstrated that *f*_H_ is often correlated with metabolic rate (e.g. Armstrong, 1986; Campbell *et al.*, 2004; Clark *et al.*, 2005; Eliason *et al.*, 2008, 2011), and thus provides an opportunity to understand the relative energetic consequences of different stressors (Cooke *et al.*, 2016).

We are aware of three studies that have applied *f*_H_ biotelemetry or biologger devices to quantify the energetic and physiological consequences of catch-and-release in salmonids. Anderson *et al.* (1998) monitored post-angling *f*_H_ recovery in Atlantic salmon (*Salmo salar*) at various temperatures; Donaldson *et al.* (2010) reported the relationship between swimming intensity, *f*_H_ and recovery in coho salmon (*Oncorhynchus kisutch*); and Raby *et al.* (2015b) measured *f*_H_ in coho salmon under various temperatures and fisheries net entanglement durations. These studies have demonstrated that fisheries interactions inflict sufficient stress to trigger a 1–2-fold increase in *f*_H_, which requires a 15–16 h recovery period to return to routine *f*_H_. Hence, these studies have shown the value of remotely measuring *f*_H_ as a proxy for metabolic rate, especially given the potential for metabolic rate dependent mortality arising from the collapse of aerobic scope (Priede, 1977), particularly at high water temperatures (Eliason *et al.*, 2011, 2013a). *f*_H_ can provide an indication of the magnitude of the fisheries stress through (1) the scope for *f*_H_, defined as the difference between peak *f*_H_ and resting *f*_H_ (2) the recovery duration, defined as the time for *f*_H_ to reach routine *f*_H_, and (3) the excess post-exercise heart beats (EPHB), defined as the number of heart beats above routine *f*_H_ that a fish spends during recovery (Raby *et al.*, 2015b).

The current study investigated Fraser River sockeye salmon *f*_H_ recovery after a simulated fisheries stress event (i.e. exhaustive exercise and air exposure) across a spectrum of ecologically relevant temperatures. This represents the first study of Pacific salmon to measure cardiac responses to exercise and air exposure intended to represent a general fisheries stressor (especially relevant to recreational fishing) and associated handling. Previous studies used a net to entangle fish (i.e. Raby *et al.* 2015b) or did not include an air exposure component nor vary temperature (i.e. Donaldson *et al.* 2010). The Fraser River is Canada’s most productive salmon migration river (Northcote and Larkin, 1989). However, recently, the average water temperature of the Fraser River has been increasing at a rate between 0.025°C and 0.044°C per year (Patterson *et al.*, 2007), reaching up to 4°C above historical means (from 1971 to 2000) in July 2015, and approaching the upper thermal threshold of some sockeye salmon populations (21°C) (Eliason *et al.*, 2011). We therefore selected Fraser River sockeye salmon as a model species due to their ecological and socio-economic relevance and the extensive research base already conducted on their physiology and temperature tolerance (i.e. Eliason *et al.*, 2011; Patterson *et al.*, 2016) that enabled us to contextualize our work. We surgically implanted *f*_H_ loggers into adult sockeye salmon, subjected them to the fisheries stressor, then allowed them to recover at three temperature regimes, one close to the optimal temperature for aerobic scope (ToptAS, 16°C) and two that approached the upper functional thermal tolerance (Tcrit, 19°C, 21°C) for 48 h while *f*_H_ was continuously monitored. We hypothesized that recovery from exhaustive exercise would be impeded at elevated temperatures. More specifically, we predicted that both the scope for *f*_H_ and the duration of recovery would be greater at supraoptimal temperatures.

## Methods

This experiment was conducted under Canadian Council on Animal Care guidelines in accordance with the standards set by Carleton University (AUP #103 128).

### Animal collection and care

Between 14 and 15 August 2015, 80 summer-run sockeye salmon (mean mass of 2185 ± 8 g) were beach seined from the mainstem of the Fraser River near Hope, British Columbia, Canada (Peters Band Land; 49.3858°N, 121.4419°W). The summer run consists of co-migrating populations (e.g. Chilko, Quensel, Stellako and others) and the number of individuals per population in the migration run varies between years (Burgner, 1991; Hodgson *et al.*, 2006; Lapointe *et al.*, 2003). Stock composition was not determined for the individual fish in the present study. However, 20 individuals sampled concurrently with the present study on 13 August 2015, consisted predominantly of sockeye salmon from the Chilko stock (*n* = 11), and also included sockeye salmon from Great Central (*n* = 1), Harrison (*n* = 2), Nahatlatch (*n* = 1), Seymour (*n* = 2), Stellako (*n* = 2), and one that could not be identified. Furthermore, the DFO stock management records from 12 to 16 August 2015, which also provides DNA samples from the same cohort as the individuals used in the present study, show that the summer run sockeye salmon stock composition consisted of predominantly Chilko and Quesnel sockeye salmon. Specifically, the stock composition per day over the 4-day sampling period consisted of 4.75% ± 2% Harrison Widgeon stock, 13.75% ± 1% Late Stuart Stellako stock, 60.75% ± 1% Chilko Quesnel stock, and 2.75% ± 1% Raft North Thompson stock (samples taken from Cottonwood, which is 4 days downstream from Peters Band Land, and at Whonoock, which is 2 days downstream from Peters Band Land) (Department of Fisheries and Oceans, 2015).

Fish were transported less than 1 h by truck to the Department of Fisheries and Oceans Canada (DFO) Cultus Lake Laboratory using 1 250 L transportation containers. Salmon were then transferred into two 22 000 L tanks that were supplied by flow-through lake water (Cultus Lake; 12.5°C ± 1°C; DO = 85–100%) under a natural photoperiod (~14 h daylight; 10 h dark). Sockeye salmon remained in the holding tank for a minimum of 48 h before any surgical manipulations. Each tank held ~40 sockeye salmon.

### Surgical procedures

A total of 67 sockeye salmon were implanted with commercially available *f*_H_ loggers (DST milli HRT, 13 mm × 39.5 mm, Star-Oddi, Iceland; http://www.star-oddi.com/) programmed to record *f*_H_ every 5 min at 200 Hz based on previous work (Clark *et al.*, 2010; Eliason *et al.*, 2013a). Raw electrocardiogram (ECG) traces were measured every 3 h to evaluate the quality of the *f*_H_ measurements.

Prior to logger implantation, salmon were anesthetized with a buffered (NaHCO_3_; 200 mg/L) tricaine methanesulfonate (MS-222; 100 mg/L) solution (Raby *et al.*, 2015b). Once equilibrium was lost, fish were promptly transferred to a surgical trough and maintained on a weaker anesthetic solution (70 mg/L MS-222; 140 mg/L NaHCO_3_), which was continuously pumped over the gills throughout the procedure.

A 5 cm incision was made on the mid-line of the ventral surface just posterior to the pectoral girdle. Loggers were inserted immediately posterior to the pericardial membrane and were sutured to the body wall (PDS II polydioxanone suture; violet monofilament, 2-0). The incision was then closed using three to four single interrupted sutures. A passive integrated transponder (PIT) tag (Oregon RFID 32 mm HDX) was injected into the dorsal musculature posterior to the dorsal fin. Fish recovery was aided by ventilating the gills with fresh water before returning it into the treatment tank (1200 L; DO = 80–100%; 12.5°C ± 1°C) for 48 h, alongside three other sockeye salmon that underwent the same procedure.

### Experimental procedures: acclimating to treatment temperature (Day 3)

Beginning 48 h after logger implantation, the water in each tank was increased by 1°C per hour until treatment temperature was reached: 16°C optimal temperature for aerobic scope, 19°C, and 21°C upper functional thermal tolerance limit (Eliason *et al.*, 2011). Temperatures were maintained at ±0.5°C from treatment target throughout the study. Since the 21°C treatment was decided later into the study, this treatment only began 13 days since the fish were brought to the lab. Otherwise, tank temperature was allocated such that treatments were distributed throughout the 3-week experimental period.

Salmon were then left to acclimate to the new temperature for 24 h. This brief acclimation period is ecologically relevant because Fraser River sockeye salmon often encounter warm water and fisheries pressure simultaneously, ~24 h after initiating upriver migration (Gale *et al.*, 2011).

### Simulated fisheries capture stress and terminal fish sampling (Day 4)

The fisheries simulation occurred ~80 h post-surgery, which exceeds the 40–72 h post-surgery recovery time reported in previous salmonid research (Anderson *et al.* 1998; Donaldson *et al.* 2010; Raby *et al.* 2015b). At mid-day (~80 h post-surgery), tagged acclimated fish were individually chased, in a separate sampling tank (500 L; doughnut-shaped) of the same temperature as their treatment tank. Salmon were dip netted from the treatment tank into the chase tank (~2 s air exposure) and chased for 3 min (Robinson *et al.*, 2013). Chasing consisted of three to four experimenters leaning over the edge of the tank and alternating waving their hands at the fish, lightly pinching the caudal fin, or splashing vigorously whenever the sockeye salmon passed by. The goal of the chase was to exhaust the fish to a similar level that would occur during a seine net, gill net, or angling event. There was no injury aspect to this chase method, nor were the chasers attempting to harm the fish in any way besides exhausting it. Similar chase methods have been applied in previous work (Milligan, 1996; Robinson *et al.*, 2013) and techniques have been refined to reasonably simulate fisheries exercise (Gale *et al.*, 2011; Cooke *et al.*, 2013). At the end of the 3 min, fish were dip netted and lifted out of the water for a 1 min air exposure (Robinson *et al.*, 2013). During this time, the fish PIT tag ID (Oregon RFID PIT tag reader) was recorded. Sockeye salmon were then returned to the original treatment holding tank and left for 48 h. *f*_H_ monitoring duration was selected based on past research stating that 24 h is insufficient for physiological recovery (Raby *et al.*, 2015b), while monitoring for 48 h is sufficient for the recovery of blood plasma constituents (Gale *et al.*, 2011). Temperature and dissolved oxygen (DO) were checked every 0.5–1 h using an Oxyguard dissolved oxygen meter (Handy Polaris), taking great care to minimize disturbance to the fish by making minimal noise and not standing within view of the fish.

At 48 h post-fisheries stressor, fish were sacrificed by cerebral concussion. A 2 ml blood sample was immediately taken via caudal puncture and stored on ice for hematocrit analysis. Fork length, weight, sex and physical condition (including an autopsy for internal condition) were recorded at that time.

Hematocrit was determined using heparinized capillary tubes (75 mm Drummond Hemato-Clad, ammonium heparin) and centrifuged (Clay-Adams, NJ) at 12 000 rpm for 5 min. Since this study targeted healthy sockeye salmon, only 35 sockeye salmon (52.2%) provided usable data; *N* = 13 in the 16°C treatment, *N* = 13 in the 19°C treatment and *N* = 9 in the 21°C treatment. Fish were removed from the sample size due to logger dislodgement producing false *f*_H_ records (*N* = 11), logger failure (*N* = 4), internal hemorrhaging (*N* = 3), injured liver (*N *= 2), premature mortality (*N* = 6), treatment temperature deviating from the target temperature treatment by more than 1°C for more that 3 h over the course of the experiment (*N* = 4), and poor condition indicated by low hematocrit (i.e. ≤20% according to Gallaugher and Farrell, 1998; *N* = 2).

### Data processing and statistical analysis

Routine, resting and peak *f*_H_ were determined for each individual fish. Resting *f*_H_ was calculated by taking the average of the lowest 10th percentile *f*_H_ from the *f*_H_ profile once the fish reached the experimental temperature. Routine *f*_H_ and post-stress recovery time (duration of *f*_H_ elevation until plateau) was calculated using breakpoint regression analysis (Schwarz, 2015) in RStudio (v. 3.2.3, RStudio Inc., Boston, MA, USA; https://www.rstudio.com/), starting from the fisheries capture simulation and ending 48 h post-fisheries stress simulation. We defined routine *f*_H_ as the *f*_H_ once the fish recovered from the fisheries stress (recovery curve plateaued). Peak *f*_H_ was defined as the most elevated *f*_H_ attained following the fisheries simulation. The time to reach peak *f*_H_ was measured beginning from the start of the capture simulation (Raby *et al.*, 2015b). Using these values, scope for *f*_H_ (peak *f*_H_ – resting *f*_H_) and factorial scope for *f*_H_ (peak *f*_H_ ÷ resting *f*_H_) were also determined. Mean treatment values were compared using one-way ANOVA (*α* = 0.05) in Sigmaplot (v. 11.0, Systat Software Inc., San Jose, CA, USA). Transformations including ln, reciprocal, and square-root were used to satisfy normality. Post-hoc analysis was conducted using Holm–Sidak non-parametric pairwise multiple comparison.

Finally, the integrals under the mean *f*_H_ curve at every half-hour, starting from the chase start time and ending once routine *f*_H_ was met (end of recovery time previously determined), provided the number of EPHB caused by the stressor over the recovery period (Raby *et al.*, 2015b). Total post-exercise heart beats (TEPHB, the sum of all the EPHB from the fisheries simulation to the recovery point) provided an estimate of the extra energy, above routine levels, that the salmon allocated towards recovery after a stress event. TEPHB was compared between temperature treatments using a one-way ANOVA (*α* = 0.05). In addition, the cumulative increase in EPHB was calculated at every hour post-stress to describe the *f*_H_ recovery profiles. This provided a quantifiable measurement for the comparison of the duration that temperature influences the rate of recovery and the added energetic cost of recovery induced by warmer water temperature after a fisheries stress event. Using RStudio and the nlme package (Pinheiro *et al.*, 2013), a linear mixed effects model was used to compare the hourly cumulative EPHB increase during recovery, treating individual fish as a random effect to correct for repeated measures of *f*_H_. This described the shape of the *f*_H_ recovery profile across temperature treatments. The model was compared using a one-way ANOVA (*α* = 0.05) and a Bonferroni adjusted Tukey *post-hoc* analysis.

## Results

### Capture simulations

**Figure 1:**
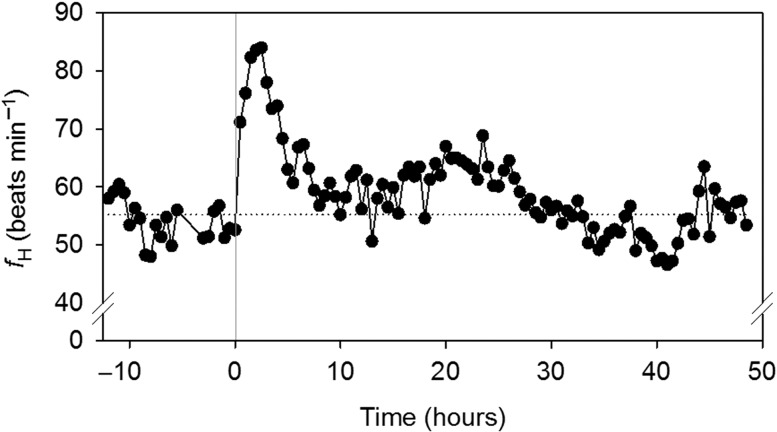
Heart rate (*f*_H_) trace of an individual sockeye salmon before, during, and after fisheries pressure simulation. Sockeye salmon was exposed to the 16°C temperature treatment. Dashed line represents the routine *f*_H_. The gray line represents the fisheries capture simulation start at Time 0.

**Figure 2:**
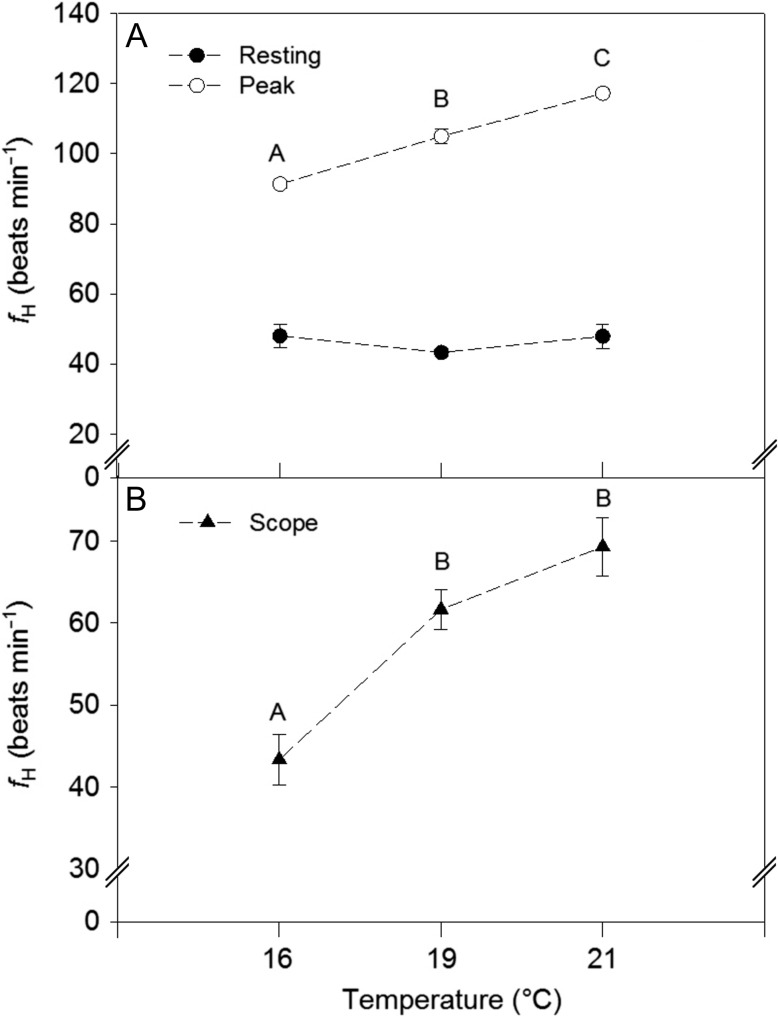
Mean (±SE) (A) resting heart rate (*f*_H_) and peak *f*_H_ and (B) scope for *f*_H_ (peak *f*_H_ – resting *f*_H_) of sockeye salmon during a fisheries capture simulation, while exposed to 16°C (*N* = 13), 19°C (*N* = 13) and 21°C (*N* = 9) water temperatures. Differing letters indicate significant differences among temperature treatments (One-way ANOVA; *P*-value <0.05).

There was a significant effect of temperature on sockeye salmon *f*_H_ during the fisheries capture simulation. Although resting *f*_H_ did not differ between treatments (16°C = 48.0 ± 3.2 beats min^−1^; 19°C = 43.3 ± 1.2 beats min^−1^; 21°C = 47.9 ± 3.0 beats min^−1^; *P*-value = 0.461; Figs. 1 and 2A), *f*_H_ traces varied with water temperature following the fisheries capture simulation. Peak *f*_H_ increased with temperature between all three treatments (*P*-value = <0.001). Sockeye salmon in the 21°C treatment experienced the highest peak *f*_H_ (117.2 ± 1.3 beats min^−1^) of the three treatments. The 19°C treatment sockeye salmon (104.9 ± 2.0 beats min^−1^) had an intermediate peak *f*_H_ and the lowest peak *f*_H_ occurred in the 16°C treatment group (91.3 ± 1.3 beats min^−1^). Similarly, scope for *f*_H_ increased with temperature (*P*-value = <0.001; Fig. 2B). However, scope for *f*_H_ did not differ significantly between the 21°C and 19°C treatments (69.3 ± 3.6 beats min^−1^ and 61.6 ± 6.0 beats min^−1^, respectively; *P*-value = 0.092), whereas scope for *f*_H_ was ~20 beats min^−1^ less in the 16°C treatment compared to the other treatments (43.3 ± 3.1 beats min^−1^; *P*-value = <0.001 between 16°C versus 21°C and 16°C versus 19°C). The same trend arose in the factorial *f*_H_, revealing the magnitude of change in *f*_H_ response induced by the fisheries stress (*P*-value = 0.004). Once again, there was no response difference between 19°C and 21°C treatments, with *f*_H_ almost tripling in both water temperatures (factorial scope = 2.4 ± 0.1 and 2.5 ± 0.1 in 19°C and 21°C, respectively; *P*-value = 0.647). Sockeye salmon in the 16°C treatment exhibited ~20% less change in *f*_H_ than fish in the 19°C and 21°C treatments (factorial scope = 1.99 ± 0.1, *P*-values = 0.003 and 0.005 comparing 16°C versus 21°C and 16°C versus 19°C, respectively).

### Recovery

**Figure 3:**
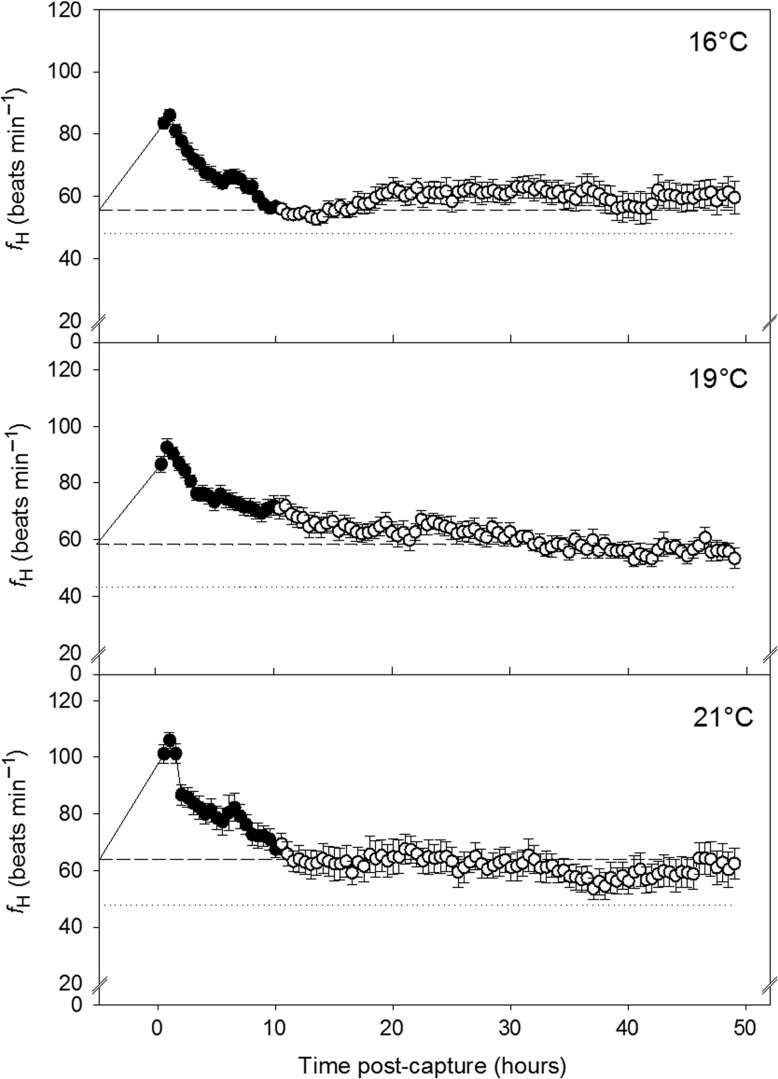
Sockeye salmon mean (±SE) heart rate (*f*_H_) recovery profiles (black circles), resting *f*_H_ (mean lowest 10th percentile of *f*_H_ from the entire experiment) (dotted line), and routine *f*_H_ (mean recovery *f*_H_ determined by breakpoint regression analysis) (dashed line) over time (hours) since fisheries capture simulation in 16°C (top), 19°C (middle) and 21°C (bottom) water. Black circles represent *f*_H_ recovery during the first 10 h of recovery. White circles show *f*_H_ recovery from 11 to 48 h post-fisheries simulation. Time 0 represents the fisheries capture simulation start time, and *f*_H_ was monitored for the next 48 h.

**Figure 4:**
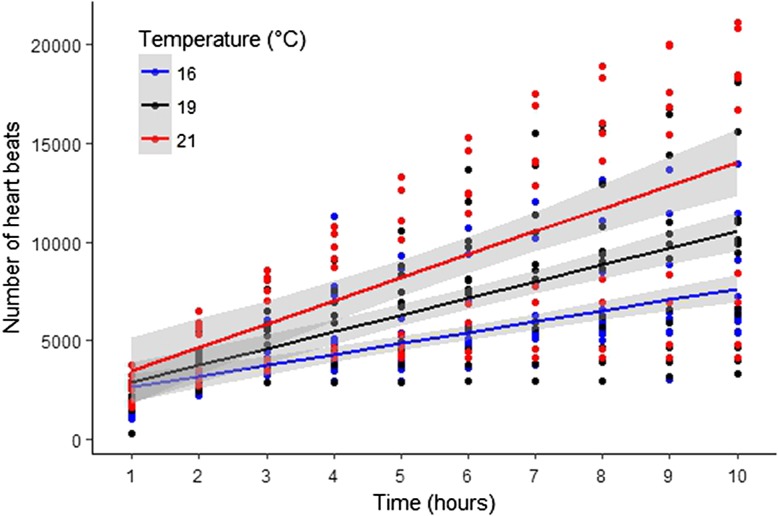
Regression plot showing the variability of cumulative excess post-exercise heart beats (EPHB) (beats) within temperature treatments, and the general relationship between temperature treatments according to the linear mixed effect model with individual fish as repeated measures, against the recovery time in hours post-fisheries capture simulation. The 16°C treatment (*N* = 13) is denoted by the blue circles and the blue regression line, the 19°C (*N* = 13) treatment is denoted by the black circles and black regression line, and the 21°C treatment (*N* = 9) is denoted by the red circles and red regression line. The shaded area around each regression line represents the 95% confidence region. Time 0 represents the start of the fisheries capture simulation.

The *f*_H_ recovery traces varied between treatment groups, where *f*_H_ decreased at different rates before reaching a plateau, implying recovery (Figs 1 and 3). For all three temperature treatments, *f*_H_ peaked at ~1 h post-capture simulation (*P*-value = 0.748), and recovered within ~10 h (*P*-value = 0.830; Fig. 3). TEPHB was highest in the 21°C treatment, but the difference was marginally non-significant at *P*-value = 0.085. Temperature strongly influenced the rate of change in EPHB during the first 10 h post-fisheries simulation (Fig. 4, time: *P*-value = <0.0001; temperature: *P*-value = 0.030). Specifically, EPHB was lower in the 16°C treatment group compared to EPHB in the 21°C treatment group (*P*-value = 0.015). The EPHB of fish exposed to 19°C did not significantly differ from either the 16°C (*P*-value = 0.470) nor the 21°C (*P*-value = 0.390) treatments. Details about when EPHB began to diverge between the 16°C and 21°C treatments remains unknown due to lack of significant interaction between temperature and time (*P*-value = 0.210).

## Discussion

Using *f*_H_ loggers, this study monitored *f*_H_ in free-swimming sockeye salmon following a fisheries simulation event. We demonstrated that water temperature mediates the *f*_H_ response to exhaustive exercise. Consistent with previous research using *f*_H_ loggers in fish (Anderson *et al.*, 1998; Donaldson *et al.*, 2010; Raby *et al.*, 2015b), *f*_H_ increased 2.0–2.5 fold with anaerobic burst exercise and recovered back to routine levels within ~10 h. Exhaustive anaerobic exercise results in a depletion of energy and oxygen stores, the disruption of ionic, osmotic and biochemical balances, hypoxemia and acidosis (Gaesser and Brooks, 1984; Wood, 1991; Scarabello *et al.*, 1992; Gale *et al.*, 2011). Recovery is fueled by an increase in aerobic metabolism (excess post-exercise oxygen consumption, EPOC) (Gaesser and Brookes, 1984; Wood, 1991; Lee *et al.*, 2003), which is supported by elevated cardiac output. The sockeye salmon in the current study exhibited the expected *f*_H_ recovery profile after the fisheries capture simulation; in all three temperature treatments *f*_H_ increased in response to the fisheries simulation and was followed by a prolonged recovery. However, the ‘rates' of *f*_H_ recovery varied with temperature though the overall recovery ‘duration' did not differ.

### Temperature effects on peak cardiac activity

Warmer water temperatures can limit cardiovascular performance and oxygen uptake (Fry, 1971). Based on the present results, fisheries interactions or exhaustive exercise occurring in higher temperatures likely influence *f*_H_ recovery in several ways. Studies have shown that high water temperature directly mediates this process by increasing the intrinsic rate of the cardiac pacemaker cells (Randall, 1970; Farrell, 1991), resulting in an increase in *f*_H_ and thus cardiac output, and thereby increasing oxygen delivery to the tissues. Yet, Eliason *et al.* (2013a) demonstrated that in swimming sockeye salmon, the rate of oxygen delivery to tissues decreases when water temperatures approach the critical maximum temperature. It has been suggested that *f*_H_ determines the upper thermal tolerance limit in sockeye salmon, where supraoptimal temperatures correspond with the scope for *f*_H_ decreasing to zero, at which point the fish experiences cardiac collapse (Brett, 1971; Eliason *et al.*, 2013a). The present study found no difference in resting *f*_H_ and routine *f*_H_ across temperature treatments, and previous work (Eliason *et al.*, 2011) suggests that all three temperature treatments could have been within the optimal thermal window for aerobic scope of these sockeye salmon (but see below). However, when high temperature was coupled with exhaustive exercise and air exposure, temperature did affect recovery. The increased peak *f*_H_, scope for *f*_H_, and factorial *f*_H_ from the 16°C to the 21°C treatment show that overall, in warmer water, sockeye salmon must have a higher *f*_H_ (and likely cardiac output and metabolic rate) to recover from exhaustive exercise (Figs 2 and 3). Similar trends have been observed in other salmonid (e.g. coho salmon; Raby *et al.*, 2015b) and non-salmonid (e.g. largemouth bass, *Micropterus salmoides*; Cooke *et al.*, 2003) fishes. Furthermore, at 21°C, sockeye salmon *f*_H_ almost attained its upper limit, reaching a peak *f*_H_ only 13 beats min^−1^ below the recorded maximum *f*_H_ of ~130 beats min^−1^ (Eliason *et al.*, 2013a). This raises concern as to whether sockeye salmon will have the metabolic and cardiac capacity to recover from exhaustive exercise (e.g. fisheries catch-and-release) in the future if water temperatures continue increasing as predicted (Patterson *et al.*, 2007).

### Factors influencing cardiac recovery

In response to the simulated catch-and-release event, sockeye salmon *f*_H_ began increasing immediately once the exhaustive exercise began, but continued to increase and peaked 1 h later (Figs 1 and 3). This trend is similar to the response observed in other studies using different salmonid species (Schreer *et al.*, 2001; Donaldson *et al.*, 2010; Raby *et al.*, 2015b). Changes in *f*_H_ are a result of differences between the relative levels of adrenergic tone and cholinergic tone (Farrell, 1993; reviewed in Reid *et al.*, 1998). Epinephrine acts on the ß-adrenoceptors on the heart, activating channels that increase the cycling of intracellular calcium, which increases the rate and force of contraction (Randall, 1970; Ask *et al.*, 1981; Ask, 1983; Farrell, 1993). Cholinergic tone counteracts the adrenergic tone by activating muscarinic receptors in the pacemaker cells causing *f*_H_ to decrease (Farrell, 1993). Furthermore, cholinergic tone increases at the start of a burst swimming event (chase) and during hypoxia (air exposure) before it becomes inhibited during exercise (Wood *et al.*, 1979; Wood and Shelton, 1980; Farrell, 1993). Exhaustive exercise exposes the heart to hypoxia and acidosis (Kiceniuk and Jones, 1977; Wood, 1991; Gale *et al.*, 2011), which may also hinder cardiac contractility (Hanson *et al.*, 2006). Therefore, during the simulated catch-and-release stress, *f*_H_ may have been initially supressed via a stronger contribution of cholinergic tone and via impaired contractility associated with a noxious venous blood environment (i.e. hypoxia and low pH). However, this response was likely followed by the release of epinephrine, increasing the contribution of adrenergic tone. Epinephrine is essential to improve cardiac contractility when the heart is exposed to hypoxia, acidosis and hyperkalemia (high [K^+^]) associated with exhaustive activity (Hanson *et al.*, 2006). We propose that over time, as the venous blood returned to normoxic levels and the relative level of adrenergic tone outcompeted the cholinergic tone, *f*_H_ increased, resulting in the observed steady increase in *f*_H_ peaking ~1 h after the catch-and-release simulation (Figs 1 and 3). However, to our knowledge, the variation in epinephrine secretion and ß-adrenoceptor activities or in acetylcholine secretion and muscarinic receptor activities have never been quantified in sockeye salmon in response to exhaustive exercise and air exposure. Further studies are required to investigate the mechanism of the delay in attaining peak *f*_H_ and the contribution of stroke volume during recovery. Given that the 1 h delay observed in the present study is consistent with exhaustive swimming experiments on Fraser summer run sockeye salmon (Eliason *et al.*, 2013b) and coho salmon (Raby *et al.*, 2015b), investigations of other Fraser River salmon, such as pink (*Oncorhynchus gorbuscha*), chum (*Oncorhynchus keta*) and Chinook salmon (*Oncorhynchus tshawytscha*), may offer insight as to whether this is a common response in all salmonids.

Although there was a significant effect of temperature treatment on *f*_H_, especially peak *f*_H_, temperature did not affect the duration of *f*_H_ recovery; sockeye salmon from all treatment groups recovered ~10 h after the catch-and-release simulation. This is two-third the recovery duration observed in other species such as Atlantic salmon (15 h at 8°C and 16.5°C; Anderson *et al.*, 1998) and coho salmon (15–16 h at 8°C Donaldson *et al.*, 2010; 15°C Raby *et al.*, 2015b). The difference in recovery duration is possibly because the salmon in this study were free-swimming. Nevertheless, reasons for the prolonged elevated *f*_H_ remains unclear especially given that metabolic oxygen consumption (MO_2_) returns to baseline within ~1–2 h post-exhaustive exercise (Eliason *et al.*, 2013b; Lee *et al.*, 2003), and the energy invested to generate a faster *f*_H_ is equivalent to the energy required for other functions, such as migrating >1 km upstream (Raby *et al.*, 2015b). However, it is known that during exhaustive exercise, cortisol, muscle pH and muscle glycogen levels increase (Milligan, 1996), while lactate, produced during anaerobic exercise, is locally metabolized in the muscle and excess lactate accumulates in the blood (Wood, 1991; Scarabello *et al.*, 1992). Past research found that rainbow trout require up to 12 h post-exercise to restore blood lactate, muscle pH and muscle glycogen concentrations back to routine levels (Stevens and Black, 1966, Milligan and Wood, 1987, Milligan, 1996), and a blood lactate threshold of 10–15 mmol L^−1^ has been proposed, beyond which repeat swim performance is impaired (Stevens and Black, 1966; discussed in Farrell *et al.*, 2000). As such, we propose that the ~10 h recovery duration in the present study may have been associated with the duration required to restore muscle pH and to complete lactate, cortisol and glycogen clearance. Alternatively, this may also show that *f*_H_ is more sensitive to exercise stress and that recovery processes are still ongoing despite MO_2_ returning to baseline, or that epinephrine remains elevated even after the exhaustive exercise event. It is also important to note that recovery duration may vary in the wild since activity (e.g. swimming against a current) promotes physiological recovery and decreases recovery duration (Farrell *et al.*, 2001; Milligan *et al.*, 2000). The fish holding tanks for our experiment had flow but not to the extent that fish had to actively swim to maintain position. Future studies are required to determine whether these trends hold in the wild.

Similar to peak *f*_H_, scope for *f*_H_ and factorial *f*_H_, all of which increase with temperature (Fig. 2), the recovery profile (during the 10 h recovery period) also differs between temperature treatments. Higher EPHB indicates greater energy allocation towards recovery (e.g. a greater slope of the recovery curve; Fig. 3). TEPHB was marginally non-significant between treatment groups likely because, irrespective of treatment, there was considerable inter-individual variation in recovery times and TEPHB (Fig. 4), and unequal variance across treatment groups that may have masked differences. It is possible, though less likely, that some fish were still recovering from surgery and this may have contributed to inter-individual variability. Alternative reasons for the observed inter-individual variability include sex-specific differences (our sample size was too small to test for sex as a factor), behavioral differences in swimming performance during the chase (i.e. fast burst swimming versus slow swimming in response to the disturbance), and individual and population differences in physiological temperature tolerance. While stock composition was not determined in the present study, it is likely that most of the fish tested were from the Chilko population based on concurrent stock analysis conducted at time of fish collection (see Methods section). Eliason *et al.* (2011) demonstrated that physiological performance (e.g. aerobic scope) varies across sockeye salmon populations. Indeed, physiological capacities and tolerances (e.g. *T*_crit_) differ even within summer-run populations (e.g. Chilko, Quesnel, Stellako). Therefore, it is possible that the effects of water temperature on sockeye salmon *f*_H_ following catch-and-release events varies between different summer-run sockeye salmon populations and this may have contributed to some of the observed variability. Future studies could investigate these possibilities further and would require significantly larger sample sizes, strategic sampling across a broader migration window, as well as appropriate budget for DNA analysis.

Regardless of the inter-individual variation, within the first 10 h of recovery, the rate of energy expenditure was highest in the 21°C group (Fig. 4). Several factors may have led to the temperature-dependent differences in EPHB rate. For example, mitochondrial oxygen demand increases with higher temperatures, necessitating a greater cardiac output to supply oxygen to the mitochondria (Farrell, 2009; Eliason *et al.*, 2013b). Furthermore, salmon in warmer water may have accumulated a greater ionic, osmotic and biochemical imbalance relative to fish in colder water. This idea is supported by the previous observation that plasma lactate was significantly higher following exhaustive exercise in sockeye salmon swum at temperatures approaching their upper functional thermal tolerance (Jain and Farrell, 2003; Eliason *et al.*, 2013b). Concurrently, oxygen uptake capacity is diminished after a simulated fisheries interaction in warmer water followed by air exposure, due to reductions in ventilation rates (Gale *et al.*, 2011). This implies that salmon in warmer water must enhance oxygen delivery via increasing *f*_H_ and stroke volume, such that EPHB is initially higher after exhaustive exercise. To a sockeye salmon migrating in the wild, it would be advantageous to minimize the duration of recovery, because swimming performance depends to a large extent on oxygen availability for aerobic scope (Brett, 1971; Omlin *et al.*, 2014). In nature, when oxygen consumption is elevated after exhaustive exercise, the amount of aerobic scope remaining for activities such as continued upstream swimming, predator avoidance, and overcoming barriers to migration (waterfalls, rapids, etc.) would be reduced (Brett, 1971; Farrell, 2009), placing the fish at risk of migration failure (Farrell *et al.*, 2008). To a semelparous, capital breeding fish like sockeye and other Pacific salmon, the implications of failed migration would equate no lifetime reproduction and to zero fitness.

## Conclusions

The current study took an experimental approach using *f*_H_ loggers to examine the combined effects of ecologically relevant temperatures and simulated fisheries capture and release on summer-run sockeye salmon physiological recovery. Although this study was framed around a fisheries event, our findings also apply to fish recovery after exhaustive exercise including burst swimming, ascending a fish ladder associated with dam passage, or crossing fast flow water, all of which are ecologically relevant situations for many salmonid and non-salmonid species of fish.

The results support the hypothesis that warm water temperatures increase physiological recovery effort after exhaustive exercise, such as a fisheries catch-and-release event. Although the overall recovery duration did not vary, warmer water temperature regimes induced a higher initial magnitude of change in *f*_H_ (i.e. peak *f*_H_) and greater rate of EPHB during the initial 10 h of recovery. Therefore, it can be concluded that sockeye salmon invest more energy in the short term (i.e. first 10 h) to recover from a fisheries stress event at higher temperatures, even at temperatures that are suspected to be within the optimal thermal tolerance window for aerobic scope. This suggests that perhaps the true optimal temperature range for physiological performance in sockeye salmon is narrower than originally thought.

Finally, the energy required to overcome a fisheries event demands energy which could otherwise be used to overcome natural migration stressors such as predation, or to maximize reproductive investment and thus fitness. This study shows that if water temperature in the Fraser River continues to increase, as is predicted, it is possible that sockeye salmon will eventually be forced to invest more energy than they can afford to overcome stressors, such that catch-and-release practices prevent successful migration or result in pre-spawning mortality. These findings support the notion that in the face of climate change, efforts to reduce stress at warmer temperatures will be necessary if selective fishing practices are to be an effective conservation strategy.
